# Acute ischemic stroke in a 7-month-old infant, risk factors, and diagnosis peculiarities: A case report

**DOI:** 10.1097/MD.0000000000017864

**Published:** 2019-11-15

**Authors:** Lorena Elena Meliţ, Cristina Oana Mărginean, Iunius Simu, Gabriela Bucur

**Affiliations:** a Department of Paediatrics; b Department of Radiology, George Emil Palade University of Medicine, Pharmacy, Science, and Technology of Targu Mures; c Emergency County Hospital, Târgu Mureş, Romania.

**Keywords:** infant, ischemic stroke, vascular malformation

## Abstract

**Introduction:**

Ischemic stroke is an extremely rare disorder in children. The timely diagnosis is essential for the outcome of these children, but unfortunately, delays in diagnosis occur frequently.

**Patient concerns:**

We report the case of a 7-month-old infant admitted in our clinic for limited movements of the superior and inferior right limbs whose onset was 27 hours before with repeated clonic movements of the right hand associated with the same manifestations in the right oral commissure lasting approximately 10 seconds.

**Diagnosis:**

The laboratory tests revealed high D-dimers, and positive IgG anti-cardiolipin and anti-beta2 glycoproteins I antibodies, whereas the genetic profile for thrombophilia revealed heterozygote mutation in *MTHFR C677T* and *A1298C genes*. Brain imaging established the diagnosis of left frontal ischemic stroke, frontal ischemic stroke, hypoplasia of internal carotid artery, and agenesia of segment M1 of median cerebral artery and segment A1 of left anterior cerebral artery.

**Intervention:**

We administered low-molecular-weight heparin, antiplatelet therapy along with vasodilators and depletive treatment, wide-spectrum antibiotics, and anticonvulsant therapy.

**Outcome:**

The neurological deficit was greatly improved, especially in the inferior limb after 6 month from the incident of stroke, and all laboratory parameters were within normal limits including the antibodies mentioned above.

**Conclusion:**

Cerebral vascular malformation, excessive weight, and altered lipid profiles contributed to the development of acute ischemic stroke in our patient.

## 1 Introduction

Stroke is defined by the association between any acute neurologic event and acute radiological abnormality with evidence of ischemia.^[1]^ Childhood stroke must occur between the age of 28 days and 18 years persisting for at least 24 hours.^[2]^ The incidence of stroke in pediatric ages is extremely low accounting for up to 3 cases in 100,000 children above 5 years of age and 8 to 13 cases per 100,000 children with the age between 5 and 14 years.^[3]^ Depending on the type of stroke, the incidence may vary between 0.3 and 2.7 cases in 100,000 children.^[4]^ Moreover, up to 90% of the children with stroke present neurologic deficits and seizures, 20% of cases present recurrences and in up to 10% is a leading cause of death.^[5,6]^ The reports of stroke in children are few in the literature and most of them originate from developed countries, this pathology remaining underdiagnosed in developing countries since data regarding this topic are nonexistent in these areas.^[7]^ Furthermore, there are multiple differences between pediatric and adult stroke. Thus, in children 50% of stroke cases are ischemic as compared to 85% of adult cases.^[8]^ The etiology is also particular in children because in comparison to adults where the most frequent causes include hypertension, atherosclerosis, and diabetes; the most commonly incriminated risk factors in pediatric patients are cardiac diseases, infections, trauma, or vascular/vasculitic and metabolic disorders.^[1,9]^

Despite the recent increasing physician's awareness regarding this life-threatening pathology in children, the timely diagnosis remains a real challenge. The diagnostic and management difficulties are related to the wide spectrum of risk factors in these ages, atypical onset, and presentation complaints, but also the lack of a consensus regarding the therapeutic approach in these patients.^[7]^ Nevertheless, in most cases, the poor outcome is related diagnostic delays due to the lack of both community awareness and symptoms recognition by medical health providers.^[7]^ Based on these striking differences, the approach of a child with stroke is different from adults. Brain imaging is mandatory for both the confirmation of an ischemic stroke and to exclude a hemorrhagic one.^[10]^ Each imagistic method has its own pros and cons. Thus, brain computed tomography (CT) presents increased sensitivity for acute hemorrhage, but low sensitivity for acute ischemic stroke within the first 6 hours, being readily available and fast. On the other hand, magnetic resonance imaging (MRI) has high sensitivity for acute ischemia, but presents other disadvantages, such as poor availability and the need for general anesthesia in children for a complete and appropriate evaluation.^[10]^ The evaluation of a child with stroke should also involve the identification of potential risk factors that might result in short-term recurrence.^[10]^ Thus, vascular imaging of the brain and neck, along with electrocardiogram, echocardiography, lipid profiles, or extended cardiac rhythm monitoring should also be taken into account in these ages.^[10]^

The acute management in case of infants and children with stroke involves appropriate oxygenation, assurance of airway integrity and adequate hydration without administering hypotonic fluids.^[10]^ In case of unknown source of ischemic stroke, anticoagulant therapy with unfractionated heparin or low-molecular-weight heparin along with antiplatelet therapy should be initiated.^[11]^ The recommendations regarding secondary prevention are controversial. Nevertheless, limited data underline that children should be administered antithrombotic agents in order to prevent acute recurrences, but the duration of this treatment is uncertain.^[12]^ The outcome is closely related to appropriateness pediatrician-child-parents relationship, and specific medical centers for children with stroke should exist also in our country in order to avoid delays in diagnosis and management.^[13]^

Our aim was to underline the particularities related to the diagnosis and management of ischemic stroke in a 7-month-old infant.

The written informed consent was obtained from the patient's mother prior to publication of this case.

## 2 Case report

### 2.1 Presenting concerns

We report the case of a 7-month-old infant admitted in our clinic for limited movements of the superior and inferior right limbs. The onset was approximately 27 hours before the admission with paroxysmal manifestations consisting in repeated clonic movements of the right hand associated with the same manifestations in the right oral commissure lasting approximately 10 seconds followed by the limitation of active movements in the superior and inferior right limbs. The family history revealed that maternal grandfather had acute myocardial infarction and maternal great grandmother had both acute myocardial infarction and stroke. His recent personal history showed an episode of acute otitis and rubella, 4 weeks before the admission and 3 weeks before the admission, respectively.

### 2.2 Clinical findings

The clinical exam at the time of admission revealed influenced general status, right central facial paresis, the lack of reaction to stimuli (no blabbing, no smiling, no sitting), paresis of the inferior and superior right limbs, flask hypotonia and reduced muscular strength at this level, a mild cardiac murmur, and excessive weight gain, weight 10.5 kg.

### 2.3 Diagnostic focus and assessment

**Figure 1:**
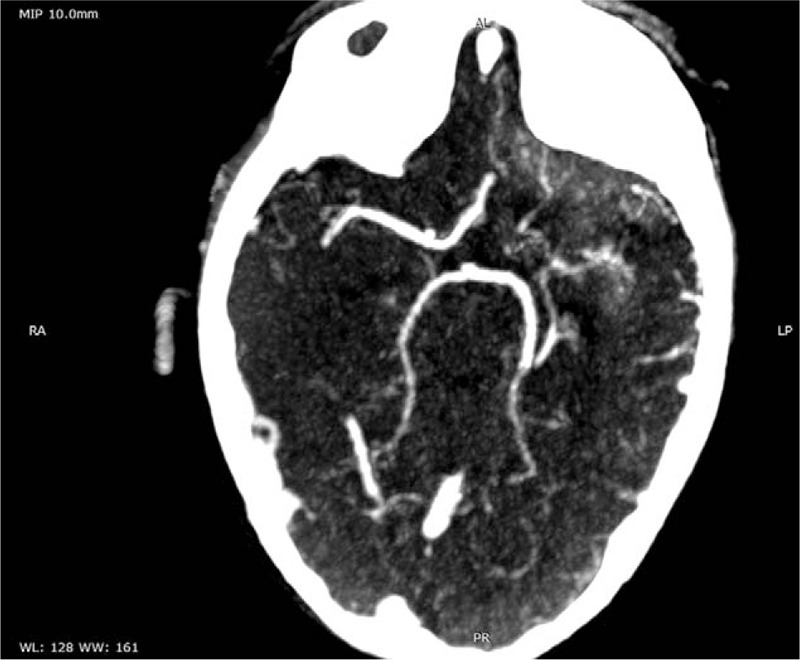
Angio CT, axial section.

**Figure 2:**
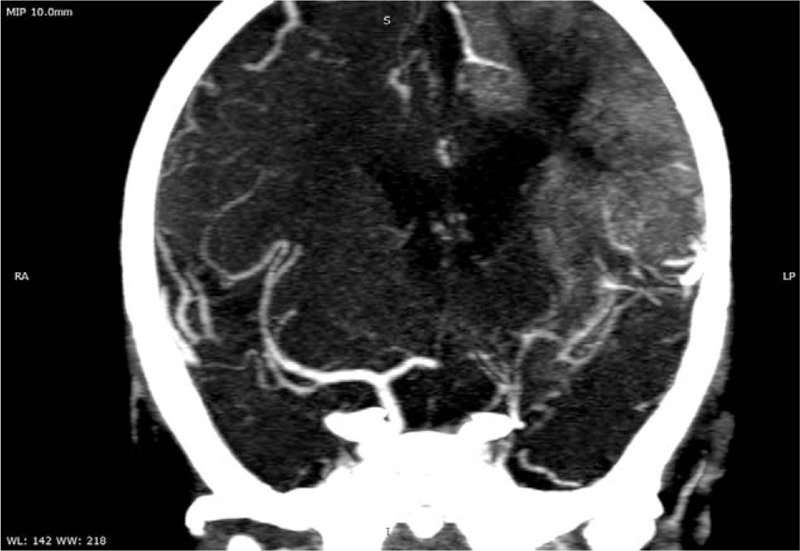
Angio CT, coronal section.

The usual laboratory tests (complete cellular blood count, inflammatory biomarkers, liver, and renal function tests) performed on admission day were within the normal ranges. The CT showed a left parietal hypodensity of approximately 20/18 mm. The brain MRI confirmed the presence of left ischemic stroke. The laboratory tests performed in order to identify potential risk factor consisted in D-dimers, anti-thrombin III, protein C and S, anti-phospholipid, anti-cardiolipin, anti-beta2 glycoproteins I, antinuclear antibodies, and the genetic profile for thrombophilia. Nevertheless, only D-dimers, IgG anti-cardiolipin and anti-beta2 glycoproteins I antibodies were positive, whereas the genetic profile for thrombophilia revealed heterozygote mutation in *MTHFR C677T* and *A1298C genes*. We also assessed the lipid profile after a 9-hours fasting period and identified hypercholesterolemia (213 mg/dl) and increased low-density lipoprotein cholesterol (137 mg/dl). The echocardiography showed a minor ostium secundum atrial septal defect and aneurism of interatrial septum, while the electrocardiogram was normal. The angio CT performed after 10 days from admission pointed out left frontal ischemic stroke, hypoplasia of internal carotid artery, and agenesia of segment M1 of median cerebral artery and segment A1 of left anterior cerebral artery (Figs. 1 and 2). The electroencephalogram established the diagnosis of right focal convulsive syndrome.

### 2.4 Therapeutic focus and assessment

On the 1st day of admission we initiated low-molecular-weight heparin, antiplatelet therapy along with vasodilators and depletive treatment in order to diminish the acute cerebral edema. We also associated wide-spectrum antibiotics since we were not able to rule out a former infection of the central nervous system. According to the neurologist recommendations, we also administered anticonvulsant therapy.

### 2.5 Follow-up and outcome

The infant is discharged after approximately 3 weeks of hospitalization with a favorable evolution and great improvements of the neurological deficit, being able to express mild active movements of both superior and inferior right limbs. After approximately 6 month, the patient presented major improvements of the neurological deficit, especially in the inferior limb, and all above-mentioned antibodies positive at onset, turned out within normal ranges. In addition, the lipid profile was normal. We also repeated the angio CT that confirmed the presence of the previously mentioned vascular malformation and showed cerebral atrophy as a result of ischemic stroke. Based on the complex cerebral vascular malformation we recommended continuing the anticoagulant therapy lifelong.

## 3 Discussions

Ischemic stroke is extremely rare in children beyond the neonatal period.^[10]^ It is well-documented that during the neonatal period, the incidence of stroke may vary between 1 in 4400 and 1 in 7700 live births, and it can also occur during the intrauterine life.^[10,12]^ Moreover, in the USA the prevalence of cerebral palsy account for 3.1 cases in 1000 children with the age of 8 years, of which up to 35% express unilateral spasticity.^[14]^ Furthermore, 89% of strokes during childhood occur in low- and middle-income countries representing a real social burden for these nations.^[15]^ Similarly, our country might be considered a middle-income nation, even though our infant was from a family with a normal socio-economic level. The most common risk factors for stroke in children are arteriopathy, cardiac diseases, other chronic disorders (e.g. iron deficiency anemia, sickle cell anemia, different genetic syndromes, aneurysms, autoimmune disorders, prothrombotic state, etc.), sepsis, shock, dehydration, and others.^[10,16]^ Of these risk factors, we identified only isolated foramen ovale in our patient, but also a complex cerebral vascular malformation. Despite the fact that dyslipidemia and obesity are known as risk factors in adults, our infant was identified with high levels of cholesterol and low-density lipoprotein cholesterol associated with excessive weight for his age, 10 kg at 7 months of age. According to recent studies that focused on identifying the factors that influence neonatal outcome, birth weight is an important predictor of further nutritional status, with a multifactorial determinism.^[17–19]^ We must also mention that our patient's birth weight was high, 4 kg that might have influenced his further excessive weight gain during the first 7 months of life. Moreover, obesity-related inflammatory status was also proved in children as well as higher lipid profiles.^[20,21]^

Delays in diagnosis are due to both the rarity of ischemic stroke and atypical symptoms in pediatric patients. Thus, stroke mimics in children might consist in seizures, migraine, syncope, Bell palsy, peripheral nerve disorders, central nervous system infection, drug intoxication, or even abdominal pain associated with other suggestive symptoms, etc.^[22–24]^ Certain intoxications, such as lead poisoning should also be taken into account for the differential diagnosis of stroke in these age groups.^[25]^ Our patient presented hemiparesis, seizures, and central facial paresis. We did not experience delays in diagnosis in our case since we established the diagnosis within the first 24 hours after admission, but unfortunately, the patient presented only after 27 hours from onset.

Neuroimaging of the brain is the most important diagnostic tool in both ischemic and hemorrhagic strokes independently of the age. Nevertheless, the first step in the assessment of a child with stroke is CT since it is readily available, and it does not require sedation. MRI and angio CT might represent 2 viable options as further steps in the evaluation of these cases. Nevertheless, a study performed on 26 patients with acute stroke suggested that magnetic resonance angiography might overestimate the stenosis in medium-sized vessels, and even miss occlusion in distal small vessels.^[26]^ A large meta-analysis that focused on strokes in children, identified malformations of the large intracranial arteries consisting in irregularities, stenosis, or occlusion as the most common etiology for ischemic stroke in pediatric patients.^[27]^ In our case, the angio CT also revealed a complex vascular malformation consisting in hypoplasia of internal carotid artery, and agenesis of both segment M1 of segment M1 of median cerebral artery and segment A1 of left anterior cerebral artery.

The outcome of children with stroke depends mostly on the timely diagnosis, but the mortality in these patients remains high. Thus, a prospective study performed on 95 children with stroke from Switzerland with a follow-up period of approximately 7 years showed an overall mortality of 14%, and 6% of the patient presented a recurrent stroke. Most of the studied patients had hemiparesis and speech impairment.^[28]^ Nevertheless, neurological deficits in children might suggest also certain genetic disorders.^[29,30]^ Fortunately, our patient's outcome was very good, with major remission of the neurological deficits after 6 months from the incident of stroke.

## 4 Conclusions

Ischemic stroke in children is an uncommon pathology that is often underdiagnosed leading to life-threatening complications. Cerebral vascular malformation, excessive weight, and altered lipid profiles contributed to the development of acute ischemic stroke in our patient. The outcome of children with stroke depends mostly on the timely diagnosis.

## Author contributions

**Conceptualization:** Lorena Elena Meliţ, Cristina Oana Marginean.

**Data curation:** Cristina Oana Marginean.

**Formal analysis:** Lorena Elena Meliţ, Gabriela Bucur.

**Investigation:** Lorena Elena Meliţ, Iunius Simu, Gabriela Bucur.

**Methodology:** Lorena Elena Meliţ, Gabriela Bucur.

**Supervision:** Cristina Oana Marginean.

**Validation:** Cristina Oana Marginean, Gabriela Bucur.

**Writing – original draft:** Lorena Elena Meliţ, Cristina Oana Marginean, Gabriela Bucur.

**Writing – review & editing:** Lorena Elena Meliţ, Cristina Oana Marginean, Iunius Simu.

## References

[1] Masri A Al-Ammouri I Clinical presentation, etiology, and outcome of stroke in children: A hospital-based study. Brain Dev 2016;38:204–8. 26341288 10.1016/j.braindev.2015.08.007

[2] Lynch JK Hirtz DG DeVeber G Report of the National Institute of Neurological Disorders and Stroke workshop on perinatal and childhood stroke. Pediatrics 2002;109:116–23. 11773550 10.1542/peds.109.1.116

[3] Kissela BM Khoury JC Alwell K Age at stroke: temporal trends in stroke incidence in a large, biracial population. Neurology 2012;79:1781–7. 23054237 10.1212/WNL.0b013e318270401d PMC3475622

[4] Roach ES Golomb MR Adams R Management of stroke in infants and children: a scientific statement from a Special Writing Group of the American Heart Association Stroke Council and the Council on Cardiovascular Disease in the Young. Stroke 2008;39:2644–91. 18635845 10.1161/STROKEAHA.108.189696

[5] Chadehumbe MA Khatri P Khoury JC Seizures are common in the acute setting of childhood stroke: a population-based study. J Child Neurol 2009;24:9–12. 18923086 10.1177/0883073808320756 PMC2896819

[6] van de Port IGL Visser-Meily AMA Post MWM Long-term outcome in children of patients after stroke. J Rehabil Med 2007;39:703–7. 17999008 10.2340/16501977-0109

[7] Yock-Corrales A Varela-Bulgarelli F Barboza C Presentation of acute childhood stroke in a tertiary pediatric emergency department. Pediatr Emerg Care 2018;34:552–7. 27749807 10.1097/PEC.0000000000000918

[8] Carvalho KS Garg BP Arterial strokes in children. Neurol Clin 2002;20:1079–100. vii. 12616682 10.1016/s0733-8619(02)00012-9

[9] Lanthier S Carmant L David M Stroke in children: the coexistence of multiple risk factors predicts poor outcome. Neurology 2000;54:371–8. 10668698 10.1212/wnl.54.2.371

[10] Lo WD Kumar R Arterial ischemic stroke in children and young adults. Continuum (Minneap Minn) 2017;23 (1 Cerebrovasc Dis):158–80. 28157749 10.1212/CON.0000000000000438

[11] Monagle P Chan AKC Goldenberg NA Antithrombotic therapy in neonates and children: Antithrombotic Therapy and Prevention of Thrombosis, 9th ed: American College of Chest Physicians Evidence-Based Clinical Practice Guidelines. Chest 2012;141 2 Suppl:e737S–S41. 22315277 10.1378/chest.11-2308 PMC3278066

[12] Grunt S Mazenauer L Buerki SE Incidence and outcomes of symptomatic neonatal arterial ischemic stroke. Pediatrics 2015;135:e1220–8. 25896840 10.1542/peds.2014-1520

[13] Mărginean CO Meliţ LE Chinceşan M Communication skills in pediatrics - the relationship between pediatrician and child. Medicine (Baltimore) 2017;96:e8399. 29069036 10.1097/MD.0000000000008399 PMC5671869

[14] Christensen D Van Naarden Braun K Doernberg NS Prevalence of cerebral palsy, co-occurring autism spectrum disorders, and motor functioning - Autism and Developmental Disabilities Monitoring Network, USA, 2008. Dev Med Child Neurol 2014;56:59–65. 24117446 10.1111/dmcn.12268 PMC4351771

[15] Feigin VL Forouzanfar MH Krishnamurthi R Global and regional burden of stroke during 1990-2010: findings from the Global Burden of Disease Study 2010. Lancet 2014;383:245–54. 24449944 10.1016/s0140-6736(13)61953-4 PMC4181600

[16] Meliţ LE Mărginean CO Georgescu A Complications of sepsis in infant. A case report. J Crit Care Med (Targu Mures) 2016;2:96–9. 29967846 10.1515/jccm-2016-0012 PMC5939135

[17] Mărginean C Mărginean CO Iancu M The role of TGF-(1 869 T>C and PPAR (2 34 C>G polymorphisms, fat mass, and anthropometric characteristics in predicting childhood obesity at birth: a cross-sectional study according the parental characteristics and newborn's risk for child obesity (the newborns obesity's risk) NOR study. Medicine (Baltimore) 2016;95:e4265. 27442659 10.1097/MD.0000000000004265 PMC5265776

[18] Mărginean CO Mărginean C Bănescu C The relationship between MMP9 and ADRA2A gene polymorphisms and mothers-newborns’ nutritional status: an exploratory path model (STROBE compliant article). Pediatr Res 2019. 10.1038/s41390-019-0347-2 PMC6760549 30791043

[19] Mărginean C Bănescu CV Mărginean CO Glutathione S-transferase (GSTM1, GSTT1) gene polymorphisms, maternal gestational weight gain, bioimpedance factors and their relationship with birth weight: a cross-sectional study in Romanian mothers and their newborns. Rom J Morphol Embryol 2017;58:1285–93. 29556619

[20] Mărginean C Meliţ L Ghiga D Early inflammatory status related to pediatric obesity (STROBE compliant article). Front Pediatr 2019;7. 31275906 10.3389/fped.2019.00241 PMC6591428

[21] Mǎrginean CO Mǎrginean C Meliţ LE New insights regarding genetic aspects of childhood obesity: a minireview. Front Pediatr 2018;6:271. 30338250 10.3389/fped.2018.00271 PMC6180186

[22] Mackay MT Chua ZK Lee M Stroke and nonstroke brain attacks in children. Neurology 2014;82:1434–40. 24658929 10.1212/WNL.0000000000000343

[23] Meliţ LE Mărginean CO Mocanu S A rare case of iron-pill induced gastritis in a female teenager: a case report and a review of the literature. Medicine (Baltimore) 2017;96:e7550. 28746201 10.1097/MD.0000000000007550 PMC5627827

[24] Mărginean MO Mărginean CO Meliţ LE The impact of host's genetic susceptibility on Helicobacter pylori infection in children. Medicine (Baltimore) 2017;96:e7612. 28746216 10.1097/MD.0000000000007612 PMC5627842

[25] Mărginean CO Meliţ LE Moldovan H Lead poisoning in a 16-year-old girl: a case report and a review of the literature (CARE compliant). Medicine (Baltimore) 2016;95:e4916. 27661040 10.1097/MD.0000000000004916 PMC5044910

[26] Husson B Rodesch G Lasjaunias P Magnetic resonance angiography in childhood arterial brain infarcts: a comparative study with contrast angiography. Stroke 2002;33:1280–5. 11988604 10.1161/01.str.0000014504.18199.0d

[27] Gumer LB Del Vecchio M Aronoff S Strokes in children: a systematic review. Pediatr Emerg Care 2014;30:660–4. 25186513 10.1097/PEC.0000000000000218

[28] Goeggel Simonetti B Cavelti A Arnold M Long-term outcome after arterial ischemic stroke in children and young adults. Neurology 2015;84:1941–7. 25862797 10.1212/WNL.0000000000001555

[29] Groselj U Tansek MZ Smon A Newborn screening in southeastern Europe. Mol Genet Metab 2014;113:42–5. 25174966 10.1016/j.ymgme.2014.07.020

[30] Zerjav Tansek M Groselj U Angelkova N Phenylketonuria screening and management in southeastern Europe - survey results from 11 countries. Orphanet J Rare Dis 2015;10:68. 26025111 10.1186/s13023-015-0283-0 PMC4451731

